# Influence of social inclusion and institutional culture on students’ interactions in clinical settings

**DOI:** 10.4102/sajcd.v70i1.991

**Published:** 2023-10-13

**Authors:** Katijah Khoza-Shangase, Margo Kalenga

**Affiliations:** 1 Department of Audiology, Faculty of Humanities, University of the Witwatersrand, Johannesburg, South Africa

**Keywords:** Afrocentric, clinical training, clinical supervisors, English as an Additional Language, undergraduate, students, South Africa

## Abstract

**Background:**

Decolonisation of the Speech-Language and Hearing (SLH) professions in South Africa to be Afrocentric is a current focus. These professions continue to hold white Eurocentric English and/or Afrikaans knowledges and practices, which are reflective of the minority. As diversity of students in higher education increases, the obvious incongruency between the language of learning and teaching (English) and institutional culture of the programmes and students who use English as an Additional Language (EAL) becomes heightened.

**Objectives:**

The study’s aim was to explore the learning and social experiences of EAL undergraduate students in a South African SLH training programme, with a specific focus on students’ experiences in patient, clinical supervisor and peer interactions in clinical situations.

**Method:**

A total of 24 participants recruited through purposive sampling were included in this cross-sectional mixed-method online survey design study. Data from the survey were analysed through descriptive and thematic analysis approaches.

**Results:**

Findings reveal a less than positive impression of EAL students in the current SLH training programme as far as their clinical experiences were concerned. The institutional culture of the SLH programme was reported to be disadvantageous to EAL students. These findings raise important implications for SLH training programmes, the regulator and the country’s SLH professions as a whole.

**Conclusion:**

This study sheds light on the significant incongruency between the existing institutional culture and the increasing diversity of students, particularly those who use EAL, in South African SLH training programmes.

**Contribution:**

Findings not only illuminate the challenges but also offer a path forward towards a more inclusive and representative SLH profession in South Africa, aligned with the principles of decolonization and Afrocentrism.

## Introduction

Since the dawn of South Africa’s democracy in 1994, several sociopolitical changes aimed at equality and equity occurred. These include access to higher education by the previously excluded black students as well as the granting of official status to all South African languages where previously only English and Afrikaans enjoyed this status (Chetty, 2012; Mutepe et al., 2021; Naicker, 2016). These changes have led to an increasing number of English as an Additional Language (EAL) students in higher education in this country (Martirosyan et al., 2015), with all their diversity characteristics typical of the rainbow nation (Mutepe et al., 2021). Additionally, the South African higher education sector is seeing increased numbers of international students congruent with the influx of international residents (Mutepe et al., 2021). This demographic profile change in higher education brings with it cultural, linguistic, economic and sociopolitical diversity that calls for reviewed perspectives on teaching and learning, research, social interaction and clinical training in fields such as Speech-Language and Hearing (SLH) professions (Almurideef, 2016; Khoza-Shangase & Mophosho, 2021).

The increased diversity in higher education within this African context decreases chances of finding the dominant language of academia, language of learning and teaching (LOLT) (English), being spoken by a majority who speak it as their native language (Linake & Mokhele, 2019). This raises serious implications for the need for institutions to interrogate their language-related rigidity, especially in programmes that fall under communication sciences, such as SLH professions, where the management of language and communication difficulties are the scopes (Khoza-Shangase & Mophosho, 2018, 2021; Pillay et al., 2020). The scopes of these professions demand language proficiency for SLH students due to implications for clinical training aspects that involve communication pathology interventions, therefore determining their lifelong professional success.

Because training of SLH professions commenced during apartheid in South Africa, the training programmes are historically grounded in English and/or Afrikaans and Eurocentric ideologies, with programme and/or institutional cultures as depicted by social, academic and clinical practices aligned to these ideologies (Khoza-Shangase, 2019; Pillay & Kathard, 2015). This is incongruent with the South African population’s demographic profile which consists of majority African language speakers. This creates linguistic and cultural diversity challenges during clinical service provision for the largely English and/or Afrikaans-speaking SLH practitioners in the absence of cultural brokers and trained interpreters (Khoza-Shangase & Mophosho, 2018, 2021; Pillay et al., 2020). This challenge exists even within the training programmes where English and/or Afrikaans-speaking lecturers and clinical supervisors must train and supervise EAL students providing clinical intervention to patients who neither speak English nor come from a Western culture perspective. The use of English as a LOLT has educational advantages for the native speakers of the language and deprivation of quality education for the EAL student (Chetty, 2012; Mutepe et al., 2021). Mutepe et al. (2021) argue that this further creates very limited interaction with the LOLT, with the consequent barrier towards entering the associated cultures (Foster & Ohta, 2005), with Khoza-Shangase and Mophosho (2021) highlighting the outcome of exclusion and marginalisation. Thus, English as a LOLT within this context creates numerous social and academic challenges for students.

Language is crucial in how an individual experiences academia. An individual’s experience of the social aspect of the space (Bolderston et al., 2008; Pappamihiel, 2002; Sanner et al., 2002), their sense of belonging or exclusion and their level of participation (Khoza-Shangase, 2019) are all influenced by language. Foster and Ohta (2005) argue that without language familiarity and comfortability, it is almost impossible to enjoyably and productively partake in a particular culture. The academic space is no different, with the LOLT and language of interaction inextricably linked to institutional culture and participation (Almurideef, 2016; Martirosyan et al., 2015). Thus, within the SLH training programmes in South Africa, one can argue that there will likely be a disconnect between the EAL students and the training programmes’ culture, which raises numerous implications for their performance, participation and general experience within the training programmes. A lack of familiarity with the entrenched institutional practices means a sense of alienation for the students, resulting in social withdrawal (Bolderston et al., 2008; Khoza-Shangase, 2019). For EAL SLH students, a lack of social interaction and cultural disconnectedness may be limiting their learning ability and engagement with content as well as their clinical practicums, ultimately deteriorating the quality of their overall university experience (Archbell & Coplan, 2021).

Archbell and Coplan (2021) argue that interactions with peers and instructors are key precursors to academic success. As such, limited to no participation bears negative implications for students. Regrettably, EAL students seem to be disposed towards eschewing class discussions and avoiding engagements, thus decreasing their academic success prospects. Numerous studies report on the tendency of EAL students to distance themselves from active participation during class discussions for multiple reasons (Bolderston et al., 2008; Chetty, 2012; Hall, 2019; Mutepe et al., 2021; Sanner et al., 2002).

In Bolderston et al.’s (2008) study, EAL radiography students revealed often refraining from discussions in lectures primarily because of poor English proficiency which hampered adequate and accurate self-expression, with considerable struggles with vocabulary. These findings coincide with Chen (2015) and Hall (2019) who indicate that in most cases, EAL students are operably proficient in the social use of the language but demonstrate difficulty with academic register. In a study on EAL nursing students, Sanner et al. (2002) identified participation withdrawal to be mainly because of the international students’ thick foreign accents, leading to feelings of inadequacy when compared to their proficient counterparts and consequent withholding from providing input and posing questions in class. Similar findings were reported by Bolderston et al. (2008) and Hall (2019), with Chetty (2012) referring to this as ‘a culture of silence’.

Stemming from issues of limited vocabulary and accent differences, EAL students dread participation out of fear of being ridiculed by peers and even educators and/or supervisors in some instances (Bolderston et al., 2008; Sanner et al., 2002). Chen (2015) revealed that EAL students experienced significant anxiety during oral presentations. Although English first language (EFL) students experience a fair share of common student anxiety, their EAL peers reportedly undergo even greater stress in attempts to perform optimally with limited language and cognitive resources (Bolderston et al., 2008; Chen, 2015; Pappamihiel, 2002).

In a study investigating factors influencing EAL student acculturation to English as a LOLT, Cheng and Fox (2008) gather that acculturation is influenced by several factors, including social interaction from which support could be accessed from peers. Archbell and Coplan (2021) concur when they outline the importance of peer support in academic achievement. This interaction is, however, greatly obstructed by a lack of acceptance, or at the very least, tolerance of EAL students and their differences by non-EAL peers (Popadiuk & Marshall, 2011; Sanner et al., 2002). Bolderston et al. (2008) and Hall (2019) further report social misunderstandings between EAL students and EFL students – and their educators, due to differences in language use and tonality. Unfamiliarity with the nature of formal and socially acceptable language and register appears to be the overriding reason behind social anxiety and strained relationships with educators (Hall, 2019; Huang, 2009; Pappamihiel, 2002). These findings, however, may not be true for EAL SLH students at the South African institution where the current study was conducted. This is considering contextual differences and the types of EAL students, as the aforementioned EAL students were international students who were mainly novices in their country of study as opposed to EAL SLH students in the current study, the majority of whom are South African by birth.

In addition to theoretical learning, SLH students undergo clinical training – an added area of challenge for EAL students (Bolderston et al., 2008). Although it may be argued that EAL students, particularly those who speak the dominant languages in the country, are at a greater advantage and better positioned to provide the public culturally and linguistically appropriate services than their EFL colleagues (Khoza-Shangase & Mophosho, 2018, 2021), EAL students remain prone to difficulties in the clinical environment considering English is the language of academic and professional exchange and that the epistemological orientation of their clinical training is not Afrocentric (Khoza-Shangase & Mophosho, 2021).

In Bolderston et al.’s (2008) study on EAL radiography students, a slew of complexities was reported. English as an Additional Language students found difficulty understanding both written and verbal instructions and finding appropriate vocabulary for case notes, which meant more time and concentration were required on their part for which clinical supervisors do not always exercise patience. Moreover, students reported feeling more confident in front of patients compared to clinical supervisors, with whom they felt more intimidated, possibly due to language flexibility when conversing with patients. On the other hand, clinical supervisors were shown to often perceive EAL students as lacking initiative and being less competent than EFL students, which in turn impinged on their confidence and clinical efficiency. Bolderston’s et al. (2008) findings are supported by Attrill et al.’s (2015) findings revealing that clinical competency is significantly compromised by poor English proficiency. This, therefore, caused patients to be disadvantaged, primarily because of the students’ accent, vocabulary, writing underperformances, as well as difficulty engaging medical terminology and jargon (Attrill et al., 2015; Shakya & Horsfall, 2000).

In view of the reviewed literature, one can argue that being from the demographic profile of the majority language and culture in South Africa does not translate to an advantage when it comes to clinical practice in SLH professions. There are numerous challenges and complexities that EAL students face during training that influence their outcomes. Limited evidence on this in SLH professions in the South African context exists, thus the value of the current study investigating the learning and social experiences of EAL undergraduate students in a South African SLH training programme, with a specific focus on investigating the students’ experiences in patient, clinical educator and peer interactions in clinical situations.

## Research methods and design

Before the study was conducted, ethical clearance was secured from the non-medical branch of the university’s Human Research Ethics Committee (HREC) (protocol number: STA_2022_10). The researchers adhered to the ethical principles in the 2001 revised Declaration of Helsinki on Research with Human Subjects of 1964 by the World Medical Association (WMA). Permission to recruit students from the SLH programme was obtained from all relevant authorities.

To obtain a rich, holistic and comprehensive view and understanding of the experiences of the EAL SLH students, this study employed a cross-sectional, descriptive, qualitative design with quantitative elements – a mixed-method online survey design (Babbie & Mouton, 2005; Wright, 2005). The online survey was virtually executed in an academic context with undergraduate students in a South African university. The university’s statistics reflected a student demographic composition as follows according to the Student Headcount Enrolment of 2021: the majority of the student population (mirroring the country’s racial composition) comprises 61.89% black South Africans, 0.30% Chinese students, 3.90% mixed race, 11.25% Indians, 13.92% white people and 8.74% international students. This diverse demographic profile is continually diversifying exponentially. Additionally, the institution forms part of the six institutions in South Africa that offer the SLH programmes.

A total of 24 participants (P) were recruited through a non-probability purposive sampling strategy (Cozby, 2009). Participants had to be EAL undergraduate, second and third year, students in the SLH programme in the 2022 academic year. The years of study were believed to be relatively more senior EAL SLH students who were best suited to provide rich, layered and informed reflections of their experiences considering they have been in the SLH programme for a reasonable period, but were not close to the researcher, hence the exclusion of the first and fourth (final) year students. The sample size represented just over half (56%) of the EAL students who met the inclusion criteria in the programme. The study focused on undergraduate students because for SLH qualification and registration to practice in South Africa, training occurs at undergraduate level.

A self-developed online survey questionnaire (cf. Appendix). presented only in English, was utilised through Google Forms, where participants were able to access the form via a link. This tool had been adapted from Abrahams’ (2021) survey where the study examined ‘Transformation in SLH Professions in South Africa: Undergraduate students’ perceptions and experiences explored’. The 41-question survey, which had both open- (11 questions) and closed-ended (30 questions), covered the following five areas:

- demographic data
- academic experiences
- clinical experiences – the current paper focuses on these experiences.
- participation factors, and
- social inclusion and institutional culture.

Prior to the main study, a pilot study was conducted to test the appropriateness of the study design and the research tool (Lowe, 2019). The pilot study was conducted on two students from the target population who did not form part of the main study, and two qualified SLH practitioners. Feedback obtained from the pilot study was used to improve on the design and tool before the main study. Once the development of the survey was finalised, a link to the survey was disseminated directly to potential participants’ student emails by the registrar’s office and departmental administrators. In estimation, completion of the survey lasted no more than 30 min. The survey link remained valid for an estimated 6–8 weeks after which participants were unable to access the survey.

Data analysis comprised both quantitative and qualitative analysis methods (Creswell & Hirose, 2019), where descriptive analysis was employed in the form of descriptive statistics, and thematic analysis of responses to the open-ended questions was adopted. The thematic analysis adhered to Braun and Clarke’s (2006) six-step approach, which included: (1) familiarising oneself with the data, (2) generating initial codes, (3) searching for themes, (4) reviewing potential themes, (5) defining and naming themes and (6) producing the report.

Trustworthiness and rigour in the study were assured by ensuring that the research process had integrity and researchers were competent, that comprehensive and thorough planning occurred prior to the study, and that the methodology selected was appropriate (Fereday & Muir-Cochrane, 2006). This involved performance of a pilot study, maintenance of credibility, participating in peer debriefing and engaging in reflexivity throughout the study.

### Ethical considerations

Ethical clearance to conduct this study was obtained from the University of the Witwatersrand School of Human and Community Development Ethics Committee (No. STA_2022_10).

## Results and discussion

**FIGURE 1:**
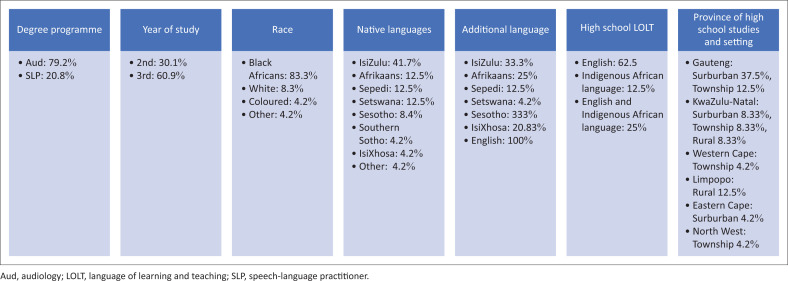
Demographic profile of participants.

The study comprised 24 EAL participants who were all females aged 18 years and over, consistent with the SLH professions profile in South Africa (Pillay et al., 2020). As depicted in Figure 1, a large percentage were native isiZulu speakers (41.7%), in line with the South African linguistic profile as isiZulu is the most widely spoken language in South Africa (Statistics South Africa, 2020). All spoken official languages, except for siSwati, Xitsonga and Tshivenda, were represented. Participants were from different provinces in South Africa, from both urban and rural areas, and were also educated in different languages at their respective schools. A large percentage was taught purely in English (62.5%), while other participants were taught in either exclusively indigenous languages (12.6%) or a mixture of an indigenous language and English (25.1%).

### Patient, clinical supervisor and peer interactions

**FIGURE 2:**
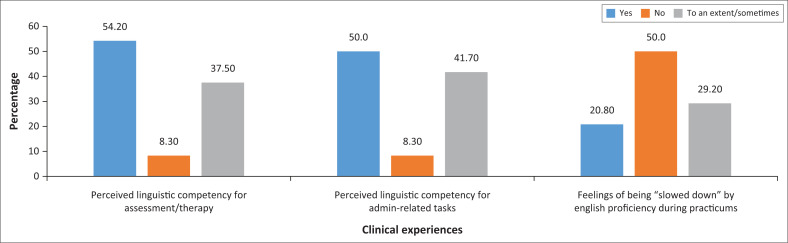
Clinical experiences of English as an Additional Language students in a Speech-Language and Hearing programme (*n* = 24).

On investigating the EAL students’ experiences in patient, clinical supervisor and peer interactions in clinical situations, as depicted in Figure 2, generally half of the participants expressed impressions of their clinical experiences that were less than positive. This was in relation to linguistic competency in completing clinical sessions (such as assessment or intervention) and administration-related clinical activities (such as record-keeping, completing forms, reporting and referral writing). Furthermore, 50% of the participants felt ‘slowed down’ by the effect of their English proficiency during their clinical practicums where completion of tasks took longer than required or expected – this happened to varying extents during clinical training. These findings are consistent with those by Bolderston et al. (2008) where EAL students reported experiencing difficulty with English proficiency when completing clinical tasks and the challenges they experienced resulted in them requiring more time and concentration, thus slowing them down. Implications are thus raised for programmes to recognise the challenges EAL students experience during the teaching and learning, and to ensure supportive measures are in place to enhance their outcomes during practicums, with supportive measures inclusive of those directed at supervisors. For example, more patience can be exercised in terms of time allocations that, for example, consider translation and back-translation; pedagogical strategies can be adapted to accommodate linguistic diversity; tools and measures used can be more diverse and inclusive; and support can be provided by clinical supervisors to assist EAL SLH students in navigating these challenges. Supervising EAL students during clinical practicums requires a thoughtful and culturally sensitive approach to ensure their success and learning. Some documented supportive measures that can be put in place to enhance the clinical experience for EAL students include clear communication and instructions, language support, interpreter services, cultural sensitivity training, peer support groups, regular check-ins, flexible evaluation, constructive feedback, cultural competence training, promote inclusivity, language and communication workshops, access to learning resources, culturally diverse patient assignments, encourage self-reflection and open communication channels (Al-Jaro et al., 2020; Felton & Harrison, 2017; Hyland & Lo, 2006; Khoza-Shangase & Mophosho, 2021; Kuo & Arcuri, 2014). By implementing these supportive measures, clinical supervisors can create an environment where EAL students can thrive, contribute effectively to patient care and develop into competent and culturally sensitive speech and language professionals.

From the open-ended questions, qualitative responses were gathered around whether being an EAL student gives the participants an advantage during their clinical practicums. As can be gleaned from the results detailed below, the majority appraised possessing an advantage over native English speakers considering the South African context in which the professions are being practiced. South Africa is a multilingual country with multilinguistic diversity and linguistic needs that need to be appropriately responded to, especially in the public hospitals or primary healthcare settings where the majority of the clientele speak an indigenous language with very limited English proficiency (Khoza-Shangase & Mophosho, 2021; Pillay et al., 2020; Seabi et al., 2014). Three themes emerged from the thematic analysis of all relevant open-ended questions: (1) ‘the EAL advantage’; (2) missing supervisors’ instructions and (3) difficulty with concise and comprehensible expression and seeming unprepared.

#### ‘The English as an Additional Language advantage’

Despite the challenges faced by EAL students, the study also identified an ‘EAL advantage’ in clinical practicums. English as an Additional Language students reported that their ability to communicate in multiple languages allowed them to build better rapport with patients and understand their experiences more effectively. Additionally, they could provide support and explanations in a way that is easily understandable to their EAL-speaking patients. This advantage is particularly important in the South African context, where linguistic diversity is prevalent, and many patients may have limited English proficiency (Seabi et al., 2014). Emphasising this advantage can lead to a more inclusive and culturally responsive approach to SLH services.

The theme of ‘the EAL advantage’ during clinical practicums identified some advantages as follows:

‘Yes, I am able to communicate with my patients using more than just one language which allows me to create a better rapport with my patient and to ensure that the information I receive is reliable, and that there is clear communication between my patient and I.’ (P2, female, SLP student)

‘Yes, because the schools we do our clinical practicals in are mostly of EAL learners therefore making communication with the learners better as I can try speak the learners’ languages.’ (P12, female, Aud student)

‘Yes it one times does, when faced with patients that are also second language English speakers it is easier to identify the gaps in their language, and if you can communicate in their language then assessment and intervention becomes easier as you would not require additional assistance (e.g., translator).’ (P13, female, SLP student)

‘Yes. I think it allows me to better understand the experiences of EAL speaking patient’s and allows me to provide better support and to explain things in a way that is easily understandable as I require the same.’ (P20, female, SLP student)

One of the participants felt that the ability to speak an indigenous language opened the lines of communication between her and her patient and assisted with building rapport, which is an important part of the patient–clinician relationship (English et al., 2022). Another participant expressed how instrumental being an EAL SLH student facilitates assessment and intervention, as an interpreter is not needed in the participant’s interactions with her EAL patients. This is particularly advantageous considering the lack of access to reliable trained interpreters in healthcare in South Africa (Mophosho, 2018; Seabi et al., 2014). In addition to this, another participant felt that being an EAL student clinician increased relatability with the patient as the student can better understand the experiences and expressions of the patient, thus better supporting them. This is perhaps attributable to the inextricable link between language and culture (Kuo & Lai, 2006). These findings indicate a shift in the linguistic and cultural inclusion of SLH services within the South African context (Khoza-Shangase & Mophosho, 2018).

Nevertheless, regardless of these perceived advantages, numerous disadvantages that couple being an EAL student in the clinical context emerged from the data, and these are reflected in the following themes.

#### Missing supervisors’ instructions

A few participants reported at times missing or misunderstanding instructions from their clinical supervisors which would impact their performance throughout the session and subsequent sessions as there would be lasting uncertainty as to what ought to be done. Below are some examples of these responses:

‘Yes, I may not understand what exactly the supervisor expects of me during my clinical sessions which may put me at a disadvantage as I will not know how to go about the rest of the clinical session or how exactly the assessment should go (including the interpretation of results) as I may have misunderstood this year to cut aspects of that specific assessment.’ (P2, female, SLP student)

‘Yes, sometimes certain instructions from supervisors contain words that I’m not often exposed to.’ (P14, female, Aud student)

‘Yes, my supervisors have only communicated to me using English and it is therefore expected of me to easily understand the information that is being communicated to me. In certain cases I may not understand what is being communicated to me […].’ (P2, female, SLP student)

The participants in the current study, as shown in the excerpts above, deemed their difficulties with English be a barrier to communication between them and their supervisors, who were mismatched linguistically and culturally for the majority of the allocations. This particular communication barrier more specifically affected instruction comprehension during clinical situations than in academic interactions. These findings support previous reports by Shakya and Horsfall (2000) who, among other challenges, cited issues related to increased failure rates among EAL students produced by the misunderstanding of general instructions, with Khoza-Shangase and Mophosho (2021) highlighting the disadvantage that EAL students providing therapy to EAL patients with an EFL clinical educator are put under. In the clinical context, the understanding of and adherence to instructions becomes even more pronounced as patients’ quality of lives are involved more than just students’ grades as in the purely academic context (Dunham et al., 2020). This then presents further implications for clinical supervisors to be attentive to EAL students and for them to also be culturally and linguistically competent in the languages of clinical supervision, if supportive measures in the form of interpreters and cultural brokers are not in place in the training programmes.

One of the challenges reported by EAL students was the difficulty in understanding instructions from their clinical supervisors, which can impact their performance during the session and lead to uncertainty in carrying out clinical tasks. The study highlights the need for clinical supervisors to be aware of the language challenges faced by EAL students and to provide clear and explicit instructions during clinical training. Effective communication between supervisors and students is crucial for successful clinical practicums and patient care.

#### Difficulty with concise and comprehensible expression and seeming unprepared

Participants also felt that they struggled with expressing themselves in a concise, understandable, academic and professional manner in clinical situations, sometimes leading to the assumption that the EAL student was incompetent or unprepared for the session. This can be seen in these excerpts:

‘[…] speaking English is not for me and I’d have to translate everything in my head before speaking and it will appear as if I’m not prepared or I don’t know what I’m talking about.’ (P5, female, Aud student)

‘[…] and it is difficult to ask for elaboration because I may be deemed as incompetent as a clinician for asking (it may be perceived as me not knowing my theory).’ (P2, female, SLP student)

‘Yes. I sometimes feel as though the way I speak makes me sound less educated, as though I struggle with sounding professional.’ (P15, female, Aud student)

From the above, it is evident that EAL students’ preparedness for clinical interaction was perceived to be questioned due to compromised expression in the LOLT. This further adds weight to previous findings in existing literature arguing that for EAL students, it is not always a matter of incompetence or lack of preparation, but rather mainly an abstract difficulty getting the words out at an appropriate standard (Bolderston et al., 2008). Furthermore, that producing the words from a thought is a process which for some involves elaborate cognitive steps of translating ideas and responses from their first language to second and/or additional language and vice versa before producing an utterance, as argued by Chen (2015). In awareness of these difficulties, implications are highlighted for clinical supervisors to identify EAL students with these challenges, provide necessary supportive measures, and attempt to better understand, assist and show the students more grace through the learning process, through the supportive strategies earlier presented (Al-Jaro et al., 2020; Felton & Harrison, 2017; Hyland & Lo, 2006; Khoza-Shangase & Mophosho, 2021; Kuo & Arcuri, 2014).

English as an Additional Language students reports of struggling with expressing themselves concisely, comprehensibly and professionally during clinical interactions could lead to perceptions of incompetence or unpreparedness among supervisors and peers. The study underscores the importance of providing support and understanding to EAL students as they navigate the linguistic challenges in clinical settings. Clinical supervisors should be trained to recognise and address these difficulties to help EAL students build their confidence and communication skills.

### Social and clinical challenges and institutional culture

When exploring the social challenges encountered by EAL SLH students during their clinical training, while also determining their perceptions of institutional culture in the SLH department, current findings confirmed that the areas of institutional culture and social inclusion were interwoven and naturally overlapped (Martinez-Acosta & Favero, 2018). An overwhelming 91.7% of the participants agreed with the suggested statement that it is possible for one to feel excluded in the SLH programme as a result of their level of English proficiency. This finding is unsurprising and is consistent with reported findings from studies in other contexts (Bolderston et al., 2008; Chetty, 2012; Sanner et al., 2002) where evidence of self-isolation of EAL students due to a lack of confidence and a sense of otherness in the face of their fluent peers was found. Although the majority expressed this possibility, over half of the participants (54.2%) indicated that they do feel represented (in terms of ethnicity, culture and language) in the academic staff members that teach them in the department. This feeling indicates progress in one of the key transformation imperatives of the university and the country, as there previously was inconsiderable representation of indigenous groups in tertiary academic staff in SLH programmes (Khoza-Shangase & Mophosho, 2018; Pillay & Kathard, 2015). However, quite contrarily, ensuing this, a large number of the participants (68.2%) reported that they felt disadvantaged by the institutional culture of the SLH programme, and a significant number (63.6%) further felt the programme is not doing enough to improve the experiences of EAL students and to help them to feel more included. Perhaps this is the result of the lack of awareness of the challenges faced by EAL students on the part of SLH programmes, or the documented resistance to transformation and decolonisation in South African higher education (Khoza-Shangase, 2019; Khunou et al., 2019).

The findings indicating that a significant number of EAL students felt excluded in the SLH programme due to their level of English proficiency indicate a sense of otherness and lack of confidence which may lead some EAL students to withdraw from engaging with their EFL peers. This withdrawal can impact peer interactions, limit peer support and potentially affect academic achievement.

The qualitative responses to the open-ended questions around social inclusion and institutional culture gave rise to the emergence of two themes: (1) withdrawal from engaging with EFL students and (2) positive relationships with relatable staff members.

#### Withdrawal from engaging with English first language students

Current authors acknowledge that social experience goes beyond the efforts of an institution or department, but more saliently involves the students with whom EAL students interact with regularly (Huang et al., 2010). Participants were asked to comment on their encounters with students who are more proficient in the LOLT. Although a few EAL students in the present study seem to socially blend in fairly well, a number of participants commented on their less pleasant social experiences in terms of interaction with EFL students, resulting in the identification of the theme of ‘withdrawal from engaging with EFL students’. The following excerpts capture this:

‘I don’t have much of an experience with them, because I prefer to spend most of my time with black students. And don’t get me wrong it’s (not) because I am racist or anything, I sometimes feel like I would be able to have conversations with them because of my proficiency in English.’ (P3, female, Aud student)

‘The experience is not that bad maybe it’s because I sometimes choose not to talk to them because I feel like I will sound stupid.’ (P5, female, Aud student)

‘I avoid talking to these students.’ (P20, female, SLP student)

‘Sometimes they’re arrogant and treat you in some type of way.’ (P23, female, SLP student)

From these responses, it is evident that there is a sense of otherness felt by EAL students, leading to withdrawal from engaging with those they perceive as more proficient in the LOLT. Thus, their overall social experience is diminished as their peer interactions are limited. This is closely related to findings by several authors who mentioned the relational dynamics between EAL students and their EFL peers (Hall, 2019; Pappamihiel, 2008; Park et al., 2017). One must note that within the South African context, these relational dynamics exist in the context where the country’s majority is the minority in the academic institution, and that majority has to speak the minority’s languages – a dynamic opposite to most international contexts such as Europe and the United States. Nonetheless, Hall (2019) reported strained relationships between EAL and EFL students due to differences in language use and tonal aspects. Park et al. (2017) found that EAL students had accents different from what was normal to their EFL peers, which led to accent stereotyping and EAL students retreating into isolation from their EAL counterparts as a result. Park et al.’s (2017) findings could be what Participant 23 in the present study was referring to when they mentioned being treated in ‘some type of way’.

Pappamihiel’s (2008) participants voiced attitudes particularly similar to those of EAL students in the present study, as they expressed having tense relations with native English speakers. In response to this, they employed a strategy of avoidance of their native English-speaking classmates to manage their language anxiety, similar to Participants 3, 5 and 20 in the present study. The participants showed a preference to rather speak English with those they felt did not feel ‘superior’ to them, and this referred to their fellow EAL speaking classmates. These findings have implications for the academic performance of the EAL SLH students as well as the speed and extent to which they acculturate to the institutional culture of their department (Archbell & Coplan, 2021; Cheng & Fox, 2008). These findings also have significant implications for the EFL students who miss out on opportunities to learn from their classmates who have a wealth of cultural and linguistic diversity skills and knowledge they could be benefitting from for the benefit of the South African patient population who are reflective of the EAL students.

Archbell and Coplan’s (2021) study shows that peer interaction and support is among one of the keys to academic success, without which academic achievement becomes difficult. Furthermore, Cheng and Fox’s (2008) study highlights the importance of peer interaction in that it has direct effects on the speed and depth of acculturation to the institutional culture of an EAL student’s institution. In light of the negative consequences for all students, not just EAL students, in terms of deprivation of peer engagement and support, implications are raised for the SLH programmes to devise strategies to promote and facilitate peer engagement between EAL students and their EFL peers.

#### Positive relationships with relatable staff members

Participants were asked if they would consider returning to the SLH programme at the university where the study was conducted for their postgraduate studies in view of their undergraduate experiences. The participant group was equally divided in responses with one half (50%) negating the possibility and the other half stating that they would return for postgraduate studies in the same department. Judging from the survey, this is understandable considering how some participants did not perceive any challenges due to language proficiency in most areas of their studies in the SLH department. More optimistically, however, some participants did commend some of the academic staff members for their efforts in making the students feel at home:

‘Although there are some difficulties, lecturers/supervisors who speak the same language as me often engage using the language during direct interactions (me and lecturer/supervisor only) and provides me with a chance to then ask my questions or get clarification on certain matters.’ (P13, female, SLP student)

‘Most lectures/supervisors are inclusive and supportive […].’ (P12, female, Aud student)

In as much as peer support is necessary for the successful adjustment of a student into the academic environment, the support of supervisors and academic staff members makes an inextricably significant contribution (Mutambara & Bhebe, 2012). This is no different in the context of EAL SLH students. In fact, Huang et al. (2010) show that a positive relationship with and support from teachers is a key aspect that may have a positive impact on academic achievement. This is because students are most likely to be motivated and may seek to perform better when they perceive their supervisors, who are mostly linguistically and culturally incongruent to the EAL students, as being supportive (Huang et al., 2010). The excerpts above show that the EAL SLH students were able to engage with certain academic staff members with whom they could identify for clarity and support. This highlights the importance of a representative staffing profile so that all students can have a sense of belonging.

This study examined the experiences of EAL undergraduate students in the SLH programme during their clinical training in South Africa. The findings shed light on the challenges and advantages faced by EAL students in patient, clinical supervisor and peer interactions, as well as the social and institutional culture implications.

The results highlighted that while approximately half of the participants expressed some difficulties with their clinical experiences related to their language proficiency, there was also an ‘EAL advantage’ in clinical practicums. English as an Additional Language students reported that their multilingual abilities allowed them to establish better rapport with patients, understand their experiences more effectively and provide support in a culturally responsive manner. This finding underscores the importance of recognising and leveraging the linguistic diversity of the South African context in SLH services.

However, the study also revealed challenges faced by EAL students, including difficulties in understanding supervisors’ instructions and expressing themselves concisely and professionally. These language-related barriers could impact the students’ performance during clinical practicums and lead to perceptions of unpreparedness. To address these challenges, clinical supervisors and academic staff should be equipped with the knowledge and skills to support EAL students effectively. Moreover, the study brought attention to the social and institutional culture challenges encountered by EAL students. Many participants felt excluded in the SLH programme due to their language proficiency, leading to withdrawal from engaging with their EFL peers. This social isolation can hinder peer learning and support, ultimately affecting academic achievement. To foster an inclusive and supportive learning environment, SLH programmes should promote interactions between EAL and EFL students, recognising the value of linguistic and cultural diversity in the field.

Overall, this study emphasises the need for culturally responsive and linguistically inclusive approaches in SLH services and training programmes in South Africa. By providing targeted support, enhancing cultural competence among academic staff and creating inclusive environments, SLH programmes can better prepare EAL students to serve the linguistically diverse population of the country.

In conclusion, the findings of this study contribute valuable insights into the experiences of EAL students in the SLH programme, highlighting the importance of recognising both the challenges and advantages they bring to the field. By implementing the implications drawn from this research, South African SLH programmes can take significant steps towards enhancing the experiences and success of EAL students, ultimately improving the quality and inclusivity of SLH services provided to the diverse South African population.

Despite the valuable insights gained from this study, there are certain limitations that need to be acknowledged. Firstly, the study had a relatively small sample size of 24 participants, all of whom were female EAL undergraduate students from one SLH programme. This limited sample size might not fully represent the diverse experiences of all EAL students in South African SLH training programmes. The findings should be interpreted with caution and may not be generalisable to larger populations. Secondly, the fact that data were collected through a self-developed online survey questionnaire, which relied on participants’ self-reporting of their experiences, introduced self-report bias. Self-report data can be influenced by social desirability bias, where participants may provide responses they think are expected rather than their true experiences. This bias could affect the accuracy and reliability of the results. Thirdly, the study used a cross-sectional design, which captures data at a single point in time. Longitudinal studies, following participants over an extended period, would provide a more comprehensive understanding of the evolving experiences and challenges faced by EAL students during their clinical training. Lastly, while the study touched on social inclusion, it primarily focused on peer interactions and institutional culture. The study did not extensively explore broader social factors such as community engagement, family support or societal perceptions, which could influence EAL students’ experiences. It is essential to consider these limitations when interpreting the results and implications of this study. Future research with larger and more diverse samples, mixed-method designs and longitudinal approaches could help address these limitations and provide a more nuanced understanding of the experiences of EAL students in SLH programmes in South Africa.

## Conclusion

With sufficient evidence indicating increasing diversity in the South African higher education and SLH student body, SLH programmes’ continued naivety to the impact of English as a LOLT as well as the non-Afrocentric institutional culture on EAL students need to be challenged. Current findings revealing a less than positive impression of EAL students as far as their clinical experiences as it relates to linguistic competency in completing clinical sessions and administration-related clinical activities are crucial to note. Even in the presence of the students’ feelings of the ‘the EAL advantage’ as it relates to the country’s patient demographic profile, EAL students faced challenges that require careful attention, with programmes putting forward strategies on how to address these challenges. Current findings also highlight a need for the regulatory SLH professional board of the Health Professions Council of South Africa, in its mandate to protect the public and guide the professions, to intensify its efforts to ensure transformed SLH professions in South Africa. Lastly, current findings raise awareness of the EAL students’ challenges in these programmes, while at the same time highlighting missed opportunities of EFL students.

## Appendix

A self-developed online survey questionnaire, presented only in English, was utilised through Google Forms, where participants were able to access the form via a link.

……………………………………………………………………………………………………………………………………………………………………………………………………………………………………

EAL, English as an Additional Language; SLH, Speech-Language and Hearing.

### Demographic and background information

1. Please state your gender:

• Male
• Female
• Other

2. Please state your race:

• Black African
• White
• Indian
• Coloured
• Other

3. Are you 18 years old or older?

• Yes
• No

4. What degree programme are you registered in?

• Audiology
• Speech-Language Pathology

5. What year of study are you currently in?

• 2nd year
• 3rd year

6. What is your country of origin?
7. What is your native language?
8. Please list any other language(s) you can speak.
  _____________________________________________________
9. What part of the country did you complete your high school studies in?

• Gauteng
• Mpumalanga
• Limpopo
• Western Cape
• Eastern Cape
• Northern Cape
• Free State
• Northwest
• KwaZulu-Natal

10. What setting was it in?

• Rural
• Urban
• Township

11. What language was used to teach you in school?
12. Were you exposed to English at school, through the media, community when growing up?

• Yes
• No

13. On a scale of 1 to 5, how proficient are you in English (with 1 representing beginner-like and 5 representing native-like). Please provide a level.
  ______

### Academic challenges

14. Do you feel confident speaking in your current language of learning and teaching (English)?

• Yes
• No
• To an extent

15. Do you think your understanding of lectures is affected by your level of English proficiency?

• Yes
• No
• To an extent

16. Do you find the standard of academic writing challenging?

• Yes
• No
• To an extent

17. Do you find assignments challenging due to English proficiency?

• Yes
• No
• To an extent

18. Do you find examinations challenging due to English proficiency?

• Yes
• No
• To an extent

19. Do you think the SLH curriculum is accommodating of different levels of proficiency in English? Please elaborate.
  Yes
  No
  Elaborate__________________________________________________________________________________________________________
  _________________________________________________________________________________________________________________
  ________________________________________________________________________
20. Do you find oral presentation tasks difficult because of language challenges (e.g., viva’s)?

• Yes
• No

21. Do you think you would perform better if you were taught in your home language?

• Yes
• No

22. If you were to suggest an ideal method of teaching, learning and assessment for an EAL student, what would it be? Please describe it below.
  _________________________________________________________________________________________________________________
  _________________________________________________________________________________________________________________
  ________________________________________________________________________
23. Any other thoughts you would like to share for this section?
  _________________________________________________________________________________________________________________
  _________________________________________________________________________________________________________________
  ________________________________________________________________________

### Clinical experiences

24. Do you think being an English as an Additional Language speaker gives you an advantage during your clinical practicums? Please elaborate.

• Yes
• No
  Elaborate__________________________________________________________________________________________________________
  _________________________________________________________________________________________________________________
  ________________________________________________________________________

25. Do you think being an English as an Additional Language speaker gives you a disadvantage during your clinical practicums? Please elaborate.

• Yes
• No
  Elaborate__________________________________________________________________________________________________________
  _________________________________________________________________________________________________________________
  ________________________________________________________________________

26. Do you feel linguistically competent for clinical activities (assessment and/or therapy)?

• Yes
• No
• To an extent

27. Do you feel linguistically competent for administration-related clinical activities (record-keeping, completing forms, report-writing, referral letters, etc.)?

• Yes
• No

28. Do you feel your level of English proficiency impacts the way you interact with clinical supervisors during clinical practicums? Please elaborate.

• Yes
• No

29. Do you feel limited or slowed down by your level of English proficiency during clinical practicums?

• Yes
• No
• Sometimes

### Participation factors

30. The way you speak English (accent, grammar, vocabulary) can influence your participation in class discussions. What are your thoughts about this?
  _____________________________________________________________________________________________________________
  _________________________________________________________________________________________________________________
  ________________________________________________________________________
31. Do you think your participation is somewhat influenced by what your peers will think of your proficiency?

• Yes
• No

32. Would you participate more in class discussions if you could speak in your native language?

• Yes
• No
  Elaborate__________________________________________________________________________________________________________
  _________________________________________________________________________________________________________________
  ________________________________________________________________________

33. Any additional thoughts you might have about your experience with participation?
  ________________________________________________________________________________________________________
  _________________________________________________________________________________________________________________
  ________________________________________________________________________

### Social inclusion and institutional culture

Institutional culture refers to the collective practices, beliefs, norms, and behaviours that historically characterise an institution (Naidoo, 2017).

34. One may feel excluded in the SLH department because of their level of English proficiency. Do you agree?

• Yes
• No

35. What are your experiences with students who are more proficient in English compared to you?
  _________________________________________________________________________________________________________________
  _________________________________________________________________________________________________________________
  _________________________________________________________________________
36. Do you feel represented in the staff members that teach you in the department in terms of ethnicity, culture, and language?

• Yes
• No

37. Do you feel advantaged or disadvantaged by the institutional culture in the SLH department?

• Advantaged
• Disadvantaged

38. Has a lecturer, clinical educator or another student ever made a comment with regards to your English proficiency that you found offensive? If yes, please elaborate on the comment without mentioning names.

• Yes
• No
  _________________________________________________________________________________________________________________
  ________________________________________________________________________________________________________________
  _____________________

39. Do you think enough is done by the department to help you to feel more included?

• Yes
• No

40. Based on your experiences, would you consider returning to the SLH department at XX for your postgraduate studies (Masters, PhD)?

• Yes
• No

41. Please describe anything else that stands out for you in terms of your experience in the SLH department as an EAL speaker.
  _________________________________________________________________________________________________________________
  _________________________________________________________________________________________________________________
  ____________________
  Thank you.

## References

[CIT0001] Abrahams, F. (2021). *Transformation in Speech-Language and Hearing professions in South Africa: Undergraduate students’ perceptions and experiences explored*. Unpublished Master’s dissertation, University of Witwatersrand, Johannesburg.

[CIT0002] Al-Jaro, M.S., Asmawi, A., & Khaleel Mohammed Abdul-Ghafour, A.Q. (2020). Supervisory support received by EFL student teachers during Practicum: The missing link. *International Journal of Language and Literary Studies*, 2(4), 22–41. 10.36892/ijlls.v2i4.437

[CIT0003] Almurideef, R. (2016). *The challenges that international students face when integrating into higher education in the United States*. *Theses and Dissertations* (p. 2336). Retrieved from https://rdw.rowan.edu/etd/2336

[CIT0004] Archbell, K.A., & Coplan, R.J. (2021). Too anxious to talk: Social anxiety, academic communication, and students’ experiences in higher education. *Journal of Emotional and Behavioral Disorders*, 30(4), 273–286. 10.1177/10634266211060079

[CIT0005] Attrill, S., Lincoln, M., & McAllister, S. (2015). International students in speech-language pathology clinical education placements: Perceptions of experience and competency development. *International Journal of Speech-language Pathology*, 17(3), 314–324. 10.3109/17549507.2015.1016109 25764340

[CIT0006] Babbie, E., & Mouton, J. (Eds.). (2005). Qualitative studies. In *The practice of social research* (pp. 269–311). Oxford University Press.

[CIT0007] Bolderston, A., Palmer, C., Flanagan, W., & McParland, N. (2008). The experiences of English as second language radiation therapy students in the undergraduate clinical program: Perceptions of staff and students. *Radiography*, 14(3), 216–225. 10.1016/j.radi.2007.03.006

[CIT0008] Braun, V., & Clarke, V. (2006). Using thematic analysis in psychology. *Qualitative Research in Psychology*, 3(2), 77–101. 10.1191/1478088706qp063oa

[CIT0009] Chen, Y. (2015). *ESL students’ language anxiety in in-class oral presentations*. *Theses, Dissertations and Capstones* (p. 962). Retrieved from https://mds.marshall.edu/etd/962

[CIT0010] Cheng, L., & Fox, J. (2008). Towards a better understanding of academic acculturation: Second language students in Canadian universities. *Canadian Modern Language Review*, 65(2), 307–333. 10.3138/cmlr.65.2.307

[CIT0011] Chetty, R. (2012). The status of English in a multilingual South Africa: Gatekeeper or liberator. In *Teaching English Today. A project of The English Academy of Southern Africa* (Vol. 4). Retrieved from https://teachenglishtoday.org/index.php/2012/06/the-status-of-english-in-a-multilingual-south-africa/

[CIT0012] Cozby, P.C. (2009). *Methods in behavioral research* (10th ed.). McGraw-Hill.

[CIT0013] Creswell, J.W., & Hirose, M. (2019). Mixed methods and survey research in family medicine and community health. *Family Medicine and Community Health*, 7(2), e000086. 10.1136/fmch-2018-000086 32148709 PMC6910743

[CIT0014] Dunham, S., Lee, E., & Persky, A.M. (2020). The psychology of following instructions and its implications. *American Journal of Pharmaceutical Education*, 84(8), AJPE7779. 10.5688/ajpe7779 32934383 PMC7473227

[CIT0015] English, W., Gott, M., & Robinson, J. (2022). The meaning of rapport for patients, families, and healthcare professionals: A scoping review. *Patient Education and Counseling*, 105(1), 2–14. 10.1016/j.pec.2021.06.003 34154861

[CIT0016] Felton, K., & Harrison, G. (2017). Supporting inclusive practicum experiences for international students across the social sciences: Building industry capacity. *Higher Education Research & Development*, 36(1), 88–101. 10.1080/07294360.2016.1170766

[CIT0017] Fereday, J., & Muir-Cochrane, E. (2006). Demonstrating rigor using thematic analysis: A hybrid approach of inductive and deductive coding and theme development. *International Journal of Qualitative Methods*, 5(1), 80–92. 10.1177/160940690600500107

[CIT0018] Foster, P., & Ohta, A.S. (2005). Negotiation for meaning and peer assistance in second language classrooms. *Applied Linguistics*, 26(3), 402–430. 10.1093/applin/ami014

[CIT0019] Hall, G. (2019). *The experiences of secondary school students with English as an additional language: Perceptions, priorities and pedagogy*. British Council. Retrieved from https://www.teachingenglish.org.uk/sites/teacheng/files/J154%20ELTRA_Secondary%20Students%20Eng%20as%202nd%20Lang%20Paper_A4_FINAL_web.pdf

[CIT0020] Huang, S., Eslami, Z., & Hu, R.J.S. (2010). The relationship between teacher and peer support and English-Language Learners’ anxiety. *English Language Teaching*, 3(1), 32–40. 10.5539/elt.v3n1p32

[CIT0021] Huang, Y.W. (2009). *Listening to their voices: An in-depth study of language anxiety and cultural adjustment among Taiwanese graduate students in the United States*. Indiana University of Pennsylvania.

[CIT0022] Hyland, F., & Lo, M.M. (2006). Examining interaction in the teaching practicum: Issues of language, power and control. *Mentoring & Tutoring*, 14(2), 163–186. 10.1080/13611260500493535

[CIT0023] Khoza-Shangase, K. (2019). Intellectual and emotional toxicity: Where a cure does not appear to be imminent. In G. Khunou, E. Phaswana, K. Khoza-Shangase, & H. Canham (Eds.), *Black academic voices: The South African experience* (pp. 42–64). HSRC Press.

[CIT0024] Khoza-Shangase, K., & Mophosho, M. (2018). Language and culture in speech-language and hearing professions in South Africa: The dangers of a single story. *South African Journal of Communication Disorders*, 65(1), 1–7. 10.4102/sajcd.v65i1.594 PMC6111603 30035607

[CIT0025] Khoza-Shangase, K., & Mophosho, M. (2021). Language and culture in speech-language and hearing professions in South Africa: Re-imagining practice. *South African Journal of Communication Disorders*, 68(1), 1–9. 10.4102/sajcd.v68i1.793 PMC8252163 34082547

[CIT0026] Khunou, G., Phaswana, E.D., Khoza-Shangase, K., & Canham, H. (Eds.). (2019). *Black academic voices: The South African Experience*. HSRC Press. Retrieved from http://hdl.handle.net/20.500.11910/14010

[CIT0027] Kuo, B.C., & Arcuri, A. (2014). Multicultural therapy practicum involving refugees: Description and illustration of a training model. *The Counseling Psychologist*, 42(7), 1021–1052. 10.1177/0011000013491610

[CIT0028] Kuo, M.M., & Lai, C.C. (2006). Linguistics across cultures: The impact of culture on second language learning. *Online Submission*, 1(1), 1–10. Retrieved from https://files.eric.ed.gov/fulltext/ED496079.pdf

[CIT0029] Linake, M.A., & Mokhele, M.L. (2019). English first additional language: Students’ experiences on reading in one South African university. *e-BANGI*, 16(9), 199–208.

[CIT0030] Lowe, N.K. (2019). What is a pilot study? *Journal of Obstetric, Gynecologic & Neonatal Nursing*, 48(2), 117–118. 10.1016/j.jogn.2019.01.005 30731050

[CIT0031] Martinez-Acosta, V.G., & Favero, C.B. (2018). A discussion of diversity and inclusivity at the institutional level: The need for a strategic plan. *Journal of Undergraduate Neuroscience Education*, 16(3), A252. 30254540 PMC6153014

[CIT0032] Martirosyan, N.M., Hwang, E., & Wanjohi, R. (2015). Impact of English proficiency on academic performance of international students. *Journal of International Students*, 5(1), 60–71. 10.32674/jis.v5i1.443

[CIT0033] Mophosho, M. (2018). Speech-language therapy consultation practices in multilingual and multicultural healthcare contexts: Current training in South Africa. *African Journal of Health Professions Education*, 10(3), 145–147. 10.7196/AJHPE.2018.v10i3.1045

[CIT0034] Mutambara, J., & Bhebe, V. (2012). An analysis of the factors affecting students’ adjustment at a University in Zimbabwe. *International Education Studies*, 5(6), 244–260. 10.5539/ies.v5n6p244

[CIT0035] Mutepe, M., Makananise, F.O., & Madima, S.E. (2021). Experiences of first-year students with using English Second Language for teaching and learning at a rural based University in a democratic South Africa. *Gender and Behaviour*, 19(2), 17795–17803.

[CIT0036] Naicker, C. (2016). From Marikana to# feesmustfall: The praxis of popular politics in South Africa. *Urbanisation*, 1(1), 53–61. 10.1177/2455747116640434

[CIT0037] Pappamihiel, N.E. (2002). English as a second language students and English language anxiety: Issues in the mainstream classroom. *Research in the Teaching of English*, 36(3), 327–355.

[CIT0038] Park, E., Klieve, H., Tsurutani, C., & Harte, W. (2017). International students’ accented English—Communication difficulties and developed strategies. *Cogent Education*, 4(1), 1314651. 10.1080/2331186X.2017.1314651

[CIT0039] Pillay, M., & Kathard, H. (2015). Decolonizing health professionals’ education: Audiology & speech therapy in South Africa. *African Journal of Rhetoric*, 7(1), 193–227.

[CIT0040] Pillay, M., Tiwari, R., Kathard, H., & Chikte, U. (2020). Sustainable workforce: South African audiologists and speech therapists. *Human Resources for Health*, 18(1), 1–13. 10.1186/s12960-020-00488-6 32611357 PMC7329495

[CIT0041] Popadiuk, N.E., & Marshall, S. (2011). East Asian international student experiences as learners of English as an additional language: Implications for school counsellors. *Canadian Journal of Counselling and Psychotherapy*, 45(3), 220–239.

[CIT0042] Sanner, S., Wilson, A.H., & Samson, L.F. (2002). The experiences of international nursing students in a baccalaureate nursing program. *Journal of Professional Nursing*, 18(4), 206–213. 10.1053/jpnu.2002.127943 12244539

[CIT0043] Seabi, J., Seedat, J., Khoza-Shangase, K., & Sullivan, L. (2014). Experiences of university students regarding transformation in South Africa. *International Journal of Educational Management*, 28(1), 66–81. 10.1108/IJEM-01-2012-0017

[CIT0044] Shakya, A., & Horsfall, J.M. (2000). ESL undergraduate nursing students in Australia: Some experiences. *Nursing & Health Sciences*, 2(3), 163–171. 10.1046/j.1442-2018.2000.00050.x

[CIT0045] Statistics South Africa. (2020). *60.6 million in South Africa*. Retrieved from www.statssa.gov.za

[CIT0046] Wright, K.B. (2005). Researching Internet-based populations: Advantages and disadvantages of online survey research, online questionnaire authoring software packages, and web survey services. *Journal of Computer-mediated Communication*, 10(3), JCMC1034. 10.1111/j.1083-6101.2005.tb00259.x

